# Insights into Peptidoglycan-Targeting Radiotracers for Imaging Bacterial Infections: Updates, Challenges, and Future Perspectives

**DOI:** 10.1021/acsinfecdis.3c00443

**Published:** 2024-01-30

**Authors:** Palesa C. Koatale, Mick M. Welling, Honest Ndlovu, Mankgopo Kgatle, Sipho Mdanda, Amanda Mdlophane, Ambrose Okem, John Takyi-Williams, Mike M. Sathekge, Thomas Ebenhan

**Affiliations:** † Department of Nuclear Medicine, University of Pretoria, 0001 Pretoria, South Africa; ‡ Nuclear Medicine Research Infrastructure (NuMeRI) NPC, 0001 Pretoria, South Africa; § Interventional Molecular Imaging Laboratory, Department of Radiology, Leiden University Medical Center, 2333 ZA Leiden, The Netherlands; ∥ Department of Anaesthesia, School of Clinical Medicine, University of Witwatersrand, 2050 Johannesburg, South Africa; ⊥ Pharmacokinetic and Mass Spectrometry Core, College of Pharmacy, University of Michigan, Ann Arbor, Michigan 48109, United States; # DSI/NWU Pre-clinical Drug Development Platform, North West University, 2520 Potchefstroom, South Africa

**Keywords:** peptidoglycan, bacteria, precursor, infection, tracer development, nuclear medicine, molecular imaging

## Abstract

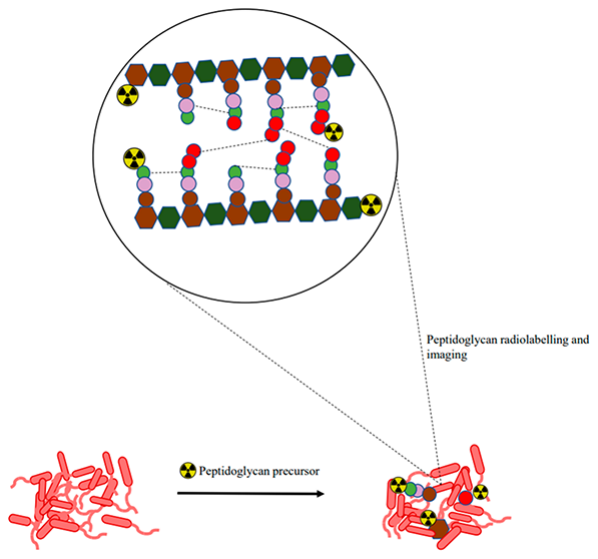

The unique structural architecture of the peptidoglycan allows for the stratification of bacteria as either Gram-negative or Gram-positive, which makes bacterial cells distinguishable from mammalian cells. This classification has received attention as a potential target for diagnostic and therapeutic purposes. Bacteria’s ability to metabolically integrate peptidoglycan precursors during cell wall biosynthesis and recycling offers an opportunity to target and image pathogens in their biological state. This Review explores the peptidoglycan biosynthesis for bacteria-specific targeting for infection imaging. Current and potential radiolabeled peptidoglycan precursors for bacterial infection imaging, their development status, and their performance *in vitro* and/or *in vivo* are highlighted. We conclude by providing our thoughts on how to shape this area of research for future clinical translation.

## 1 Introduction

Bacterial infections continue to pose the greatest threat to the human population today. This is primarily due to the emergence of antibiotic resistance, which has continued to be a global health challenge.^1,2^ It is worth noting that by the year 2050, antibiotic resistance is estimated to be among the leading causes of death, surpassing cancer, resulting in approximately 10 million deaths annually.^3^ Consequently, there is an urgent need to prioritize efforts to preserve antibiotics’ effectiveness. Achieving this goal necessitates swift action, including the prompt localization and identification of the bacterial pathogen at an early stage. Advancements in diagnostic accuracy are crucial in this regard.^4^ Microbiological cultures and the polymerase chain reaction (PCR) are routine diagnostic tools for detecting infections. However, these methods often face challenges in obtaining clinical samples, especially in the case of deeply localized infections.^5^ Furthermore, culturing pathogens is time-consuming, leading to delays in treatment, especially when dealing with pathogens that are difficult to cultivate.^6,7^

Alternatively, magnetic resonance imaging (MRI) and computed tomography (CT) offer non-invasive methods for the diagnosis of infection, particularly in musculoskeletal infections. However, these imaging techniques primarily detect anatomical abnormalities and may not always reveal the presence of an infection.^5,6^ Moreover, when it comes to early detection of infection, morphologic imaging using ultrasound (US), CT, and MRI are not well-suited, as these modalities primarily identify tissue architectural distortion that often occurs at an advanced stage of infection.^8^ To address the limitations mentioned above, nuclear medicine imaging scans such as positron emission tomography (PET) and single-photon emission computed tomography (SPECT) have been considered promising alternatives, because they can assess early infection-related physiological abnormalities.^8,9^ In hybrid imaging of infection, SPECT and PET are combined with the morphological information afforded by the anatomic imaging with CT or MRI for improved specificity.^10−12^ The most recently added imaging modality is the advanced ultrafast large axial field of view (LAFOV)PET/CT, guaranteed to provide high sensitivity at low radiation doses.^13^

These nuclear medicine imaging techniques rely on diagnostic radiotracers, with the most commonly used infection imaging agents being radiolabeled white blood cells, [^67/68^Ga]citrate, [^111^In]oxine/[^99m^Tc]hexamethylpropyleneamine oxime (HMPAO), and 2-deoxy-2-[^18^F]fluoro-d-glucose ([^18^F]FDG). However, despite their wide application in infection imaging, these radiotracers have limitations in differentiating infection from inflammation, which can lead to ambiguity in diagnosis.^12,14,15^

The lack of specificity in these radiotracers indicates that there is still an unmet need for clinically differentiating inflammation from infection. This limitation restricts the diagnostic potential that nuclear imaging tools can provide for effectively diagnosing infection.^16^ Nuclear medicine imaging technology is rapidly advancing, and improving the selectivity of bacteria-specific radiotracers will revolutionize how infections are diagnosed and treated in clinical settings.^16,17^ The ability to detect infections early offers numerous advantages, including patient-tailored antibiotic treatment, treatment response evaluation, and non-responder identification. These capabilities are challenging but crucial in tackling antibacterial resistance.^15^ To realize these possibilities, efforts have been undertaken to improve imaging specificity by developing radiotracers that target bacteria-specific biological structures and biochemical processes unique to bacteria. Some examples of these radiotracers are [^18^F]fluorodeoxysorbitol for carbohydrate metabolism, [^68^Ga]triacetylfusarinine C for iron transportation, and 1-(2-deoxy-2-fluoro-β-d-arabinofuranosyl)-5-[^125^I]iodouracil for nucleic acids.^8,18−20^ Another promising radiotracer is the ^99m^Tc- or ^68^Ga-labeled ubiquicidin fragment UBI_(29–41)_, which binds to negatively charged bacterial cell membranes and has shown specificity and effectiveness in clinical applications.^21^

**Figure 1:**
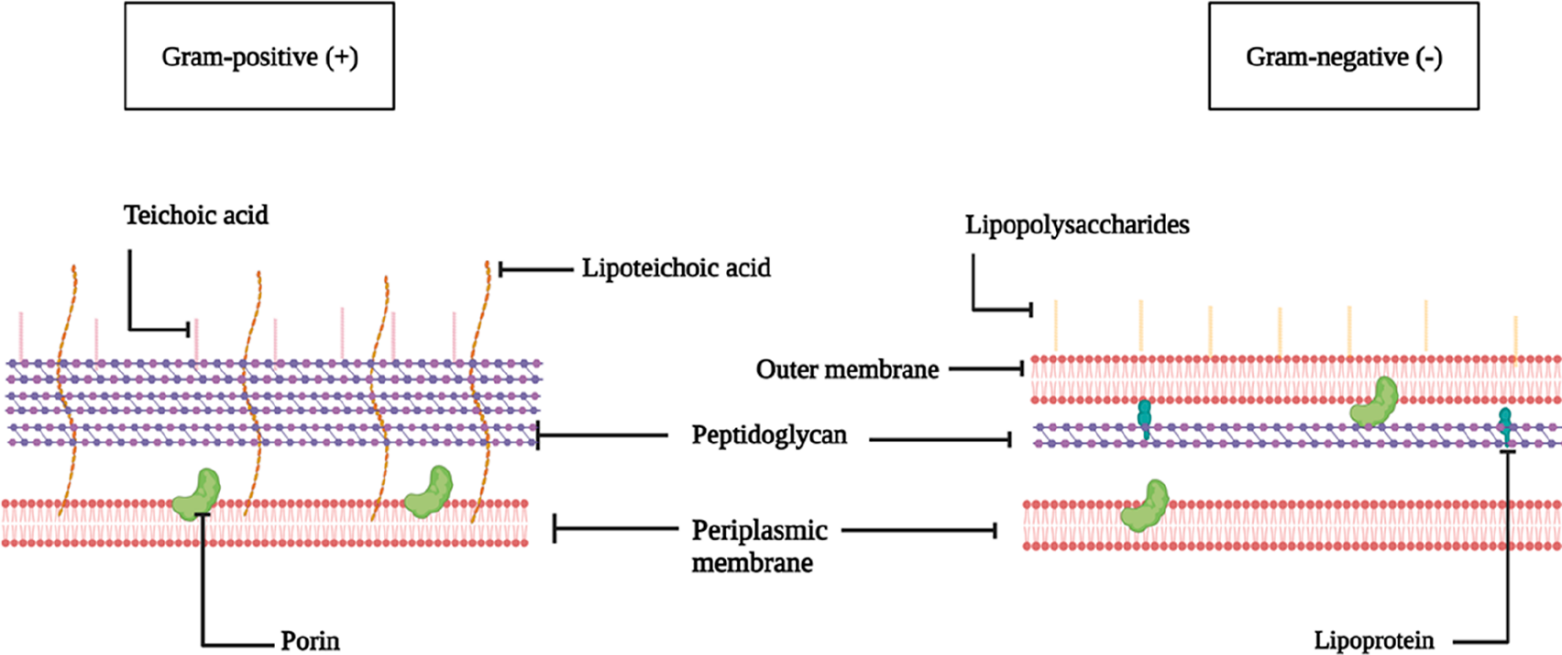
Structural architecture of Gram-positive and Gram-negative bacteria. Adapted with permission from ref (24) Copyright 2017 American Chemical Society. Created with BioRender.com.

Over the years, the distinctive structure of the bacterial cell wall has garnered significant interest, as it is absent in mammalian cells. The bacterial cell wall plays a role in the virulence and invasiveness of different bacterial strains.^22^ The Gram-negative bacterial cell wall consists of a thinner sheet of peptidoglycan meshed in between the cytoplasmic membrane and an outer membrane compared to Gram-positive bacteria. This includes the attachment of lipopolysaccharides and lipoproteins to the outer membrane. On the other hand, in Gram-positive bacteria, the cell wall consists of a thicker envelope of peptidoglycan harboring teichoic acids and lipoteichoic acids^23−26^ (Figure 1). Therefore, the uniqueness of the bacterial cell wall offers several potential targets that can be used to improve the specificity and selectivity of SPECT and PET radiotracers. The peptidoglycan layer, in particular, is a key distinctive feature of the cell wall used to identify bacteria as either Gram-negative or -positive using Gram-staining. Furthermore, it continues to serve as a cornerstone in developing target-based antibiotics.^27−30^ The heterogeneity of peptidoglycan offers the advantage of establishing whether Gram-negative or Gram-positive bacterial strains caused the infection. For this reason, it presents as an attractive target for designing radiotracers capable of accurately detecting infection with a possible identification of the causative pathogen, especially for pathogens that are difficult to cultivate or target, such as those hiding in biofilms.^23−26,31^ This is critical in conditions where multiple species of pathogens are present after initial antimicrobial therapy, as the infection may become more complex. Trying to image and treat all strains simultaneously can lead to challenges in determining the most effective treatment regimen. Hence, targeting specific strains helps simplify the approach and allows for patient-tailored antibiotic therapy, a highly advocated strategy for addressing the continuing rise of antibiotic resistance.^32^ For example, antibiotic-based radiotracers such as [^99m^Tc]vancomycin, which inhibit the peptidoglycan biosynthesis, have been studied for the selective imaging of Gram-positive bacteria. However, issues of high background activity and low sensitivity have hampered their successful translation into the clinical management of infections.^33^

The many possibilities of peptidoglycan biosynthesis targeting for bacteria-specific imaging have been vastly demonstrated with fluorescence-based peptidoglycan precursors, with recent advances in development approaches outlined in exceptionally detailed reviews.^34−37^ These reviews introduce peptidoglycan precursors as a new target of molecular imaging probes for the bacterial cell wall. Herewith, we summarize the development and applications of radiolabeled peptidoglycan precursors for SPECT and PET. In addition, examples from fluorescent-based probes will highlight unexplored peptidoglycan biochemical processes with the potential for specific targeting. We also aim to address the strategic approach to radiolabeled probe design and validation and present the current challenges hampering research into peptidoglycan targeting radiotracers for infection imaging.

## 2 Overview of Peptidoglycan Biosynthesis and Recycling Pathway as a Potential Target

**Figure 2:**
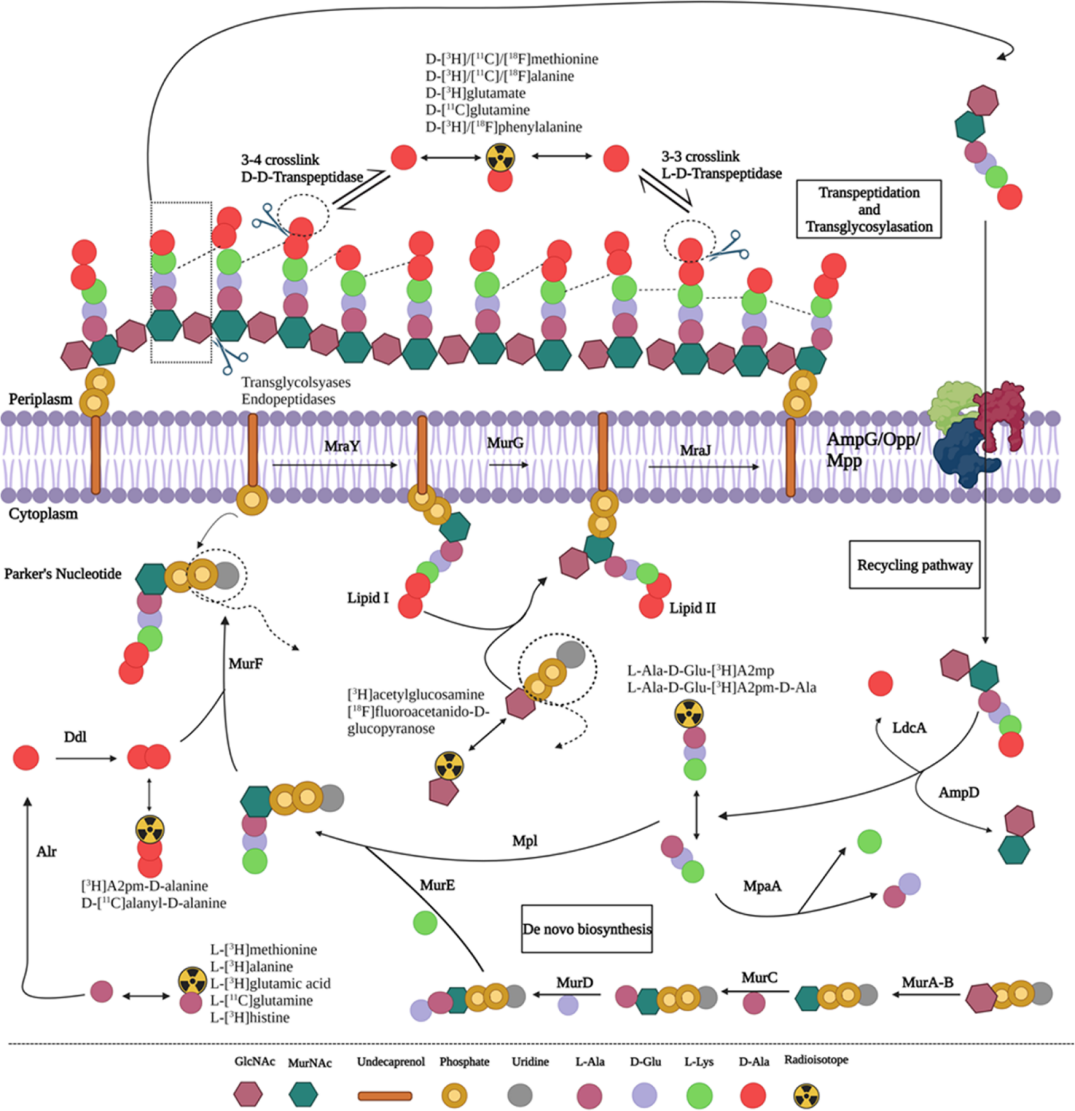
Proposed pathways of peptidoglycan biosynthesis and potential precursors for designing radiotracers or probes for imaging. Adapted from ref (34) with permission from the Royal Society of Chemistry. Created with BioRender.com.

The biochemical process of the peptidoglycan biosynthesis begins in the bacterial cytoplasm (Figure 2), where uridine diphosphate-*N*-acetylglucosamine (UDP-GlcNAc) is enzymatically transformed to UDP-*N*-acetylmuramic acid (UDP-MurNAc) by UDP-*N*-acetylglucosamine enolpyruvyl transferase (MurA) and UDP-*N*-acetylenolpyruvoyl glucosamine reductase (MurB). This reaction is followed by the sequential addition of amino acids to UDP-MurNAc to form a peptide stem of the peptidoglycan facilitated by Mur ligases. First, l-alanine (l-Ala) is covalently attached to the UDP-MurNAc via UDP-*N*-acetylmuramoyl-l-Ala ligase (MurC), followed by the addition of d-glutamate (d-Glu) in position 2 via UDP-*N*-acetylmuramoyl-l-alanine-d-glutamate ligase (MurD). Position 3 is then occupied by either *meso*-diaminopimelate (A2pm or DAP) or l-lysine (l-Lys), facilitated by UDP-*N*-acetylmuramoyl-l-alanyl-d-glutamate-2,6-diaminopimelate ligase (MurE). The addition of a d-Ala-d-Ala dipeptide substrate in positions 4 and 5 of the UDP-MurNAc-tripeptide is catalyzed by UDP-*N*-acetylmuramoyl-tripeptide-d-alanyl-d-alanine ligase (MurF) and results in the formation of a UDP-MurNAc-pentapeptide (l-Ala-γ-d-Glu-meso-A2pm/l-Lys-d-Ala-d-Ala) precursor.^38−40^

The second step occurs in the periplasmic membrane, where the phospho-*N*-acetylmuramoyl-pentapeptide transferase (MraY) attaches the UDP-MurNAc-pentapeptide to the membrane-embedded undecaprenyl phosphate carrier lipid (C55-P), resulting in the formation of C55-pyrophosphoryl (PP)-MurNAc-pentapeptide (lipid I).^41,42^ Thereafter, lipid I is further processed by *N*-acetylglucosaminyl transferase (MurG), adding a GlcNAc moiety to form a C55-PP-GlcNAc-MurNAc-pentapeptide (lipid II), followed by a subsequent flippase (MraJ) transportation to the periplasmic space.^43−45^ The third step occurs in the periplasmic space, catalyzed by bifunctional penicillin-binding proteins (PBPs). The glycosyltransferases (GTases) and transpeptidases (TPs) add lipid II to the growing peptidoglycan strand by polymerization of alternating sugar moieties^40,46^ to form a rigid peptidoglycan macromolecular layer.^39,47^

During cell elongation and proliferation, the bacteria rearrange the cell membrane by enzymatic decomposition of the peptidoglycan chain using transglycosylases and endopeptidases.^48,49^ The primary byproducts of this process include GlcNAc-anhMurNAc-tetrapeptides, which enter the cytoplasmic area using AmpG permease for re-use in peptidoglycan synthesis.^50,51^ Once inside the cytoplasm, the *N*-acetylmuramyl-l-alanine amidase (AmpD) hydrolyzes the tetrapeptide from the glycan strand.^52,53^ Subsequently, ld-carboxypeptidase (LdcA) cleaves the terminal d-Ala in position 4, resulting in a tripeptide.^54,55^ The tripeptide is then enzymatically linked to GlcNAc by murein peptide ligase (Mpl), forming a UDP-MurNAc tripeptide.^56,57^ Alternatively, the tripeptide precursor is further digested into single amino acids by murein peptide amidase (MpaA), which then re-enters the peptidoglycan biosynthesis pathway.^58,59^

Since the heterogeneity and complexity of the peptidoglycan biosynthesis and its recycling pathway involve multiple steps facilitated by the interaction of numerous precursors and enzymes, multiple strategies may be followed to develop tools for specifically targeting bacterial cell walls. In particular, the possibility for peptidoglycan precursor-based probes using different fluorophores for cell wall studies and diagnostic purposes using modern molecular techniques should be emphasized. The next part of this Review will provide an overview of radiolabeled peptidoglycan-based precursors for infection imaging and highlight potential precursors or derivatives, with examples from fluorescence imaging.

## 3 Emerging Radiotracers Targeting Peptidoglycan Biosynthesis

Table 1 summarizes the mechanism of action-based characteristics of relevant radiotracers, discussed in detail in the following sections, including their *in vitro* and *in vivo* evaluation.

**Table 1:** Mechanism of Action-Based Characteristics of Relevant Radiotracers Including Their *In Vitro* and *In Vivo* Evaluation^a^

Radiotracer	Proposed mechanism of action	Key enzymes	*In vitro* binding to pathogens	*In vivo* evaluation	Bacterial species	Comment	Refs
Amino Acid Precursors							
d-[^3^H]/[^11^C]/[^18^F]methionine	Extracellular/intracellular transpeptidation	TPs/Ddl	Uptake/competition studies	Murine myositis model d-[^11^C]Met vs l-[^11^C]Met, lung-infection model [^18^F]FDG vs d-[^3^H]Met	Broad spectrum	More sensitive than [^18^F]FDG and l-[^11^C]Met	(66−71)
				Live and/or heat-killed		Shows promise for use in clinical settings	
						Patients with suspected prosthetic joint infections	
						Low RCY with [^18^F]	
d-[^3^H]/[^11^C]/[^18^F]alanine	Extracellular/intracellular transpeptidation	TPs/Ddl	Competition studies	Murine myositis model uptake/biodistribution ([^18^F]FDG vs [^68^Ga]citrate vs d-[^11^C]Ala)	Broad spectrum	Highest accumulation observed, but low bacterial uptake after ^18^F-substitution	(67, 69, 71, 72)
				Live and/or heat-killed			
				d-Ala was further tested in vertebral discitis/osteomyelitis model, pneumonia lung infection model, and an antimicrobial therapy model			
d-[^3^H]glutamate	Extracellular/intracellular transpeptidation	TPs/Ddl	Uptake/competition studies	NA	*E. coli*	Non-specific uptake	(67)
d-[^11^C]glutamine	Extracellular/intracellular transpeptidation	TPs/Ddl	Uptake/competition studies	murine myositis model	*E. coli*	Bacterial infection imaging specificity	(75)
			Live vs heat-killed	Live or heat-killed (d-[^11^C]Gln vs l-[^11^C]Gln vs [^18^F]FDG)	*S. aureus*		
d-[^3^H]/[^18^F]phenylalanine	Extracellular/intracellular transpeptidation	TPs/Ddl	Uptake/competition studies	NA	*E. coli*	Higher uptake than [^18^F]FDG	(66, 67)
d-[^18^F]azidoalanine	Extracellular/intracellular transpeptidation	TPs/Ddl	Metabolic click chemistry assay	NA	*E. coli*, *S. aureus*	d-azidoalanine pre-targeting labeling with [^18^F]sulfo-DBCO	(123)
l-[^3^H]alanine	Intracellular racemization	Racemases	Uptake/competition studies	Myositis infection model	*E. coli*	Possible false negative results in practice	(69, 78)
l-[^3^H]methionine	Intracellular racemization	Racemases	Uptake studies	Lung-infection-model ([^18^F]FDG vs l-[^3^H]Met)	*E. coli*	More sensitive than [^18^F]FDG	(69, 78)
l-[^11^C]glutamine	Intracellular racemization	Racemases	Uptake/competition studies	Murine myositis model	*E. coli*	Poor imaging contrast and specificity	(75)
			Live vs heat-killed	Live vs heat killed (l-[^11^C]Gln vs d-[^11^C]Gln)	*S. aureus*		
l-[^3^H]glutamic acid	Intracellular racemization	Racemases	Uptake studies	NA	*E. coli*	Highest uptake in log-phase bacteria culture	(78)
l-[^3^H]histidine	Intracellular racemization	Racemases	Uptake studies	NA	*E. coli*	Highest accumulation in the stationary-phase bacteria culture	(78)

Dipeptide Precursors							
[^3^H]-A2pm-d-Ala	Intracellular peptide stem synthesis	Dld	Uptake/competition studies	NA	*E. coli*	Dipeptide degraded at the terminal before integration into cell wall	(93)
d-[^11^C]Ala-d-Ala	Intracellular peptide stem synthesis	MurF	Uptake studies, live vs heat-killed bacteria	NA	Broad spectrum	Accumulation in a wide variety of Gram(+) and (−) strains	(72)

Oligopeptide precursors							
l-Ala-d-Glu-[^3^H]A2pm	Intracellular peptide stem synthesis	Mpl	Uptake/competition studies	NA	*E. coli*	Tripeptide stable before integration into cell wall	(93)
l-Ala-d-Glu-[^3^H]A2pm-d-Ala	Intracellular peptide stem synthesis	Mpl	Uptake/competition studies	NA	*E. coli*	Tetrapeptide d-Ala moiety degraded before integration into cell wall	(93)

Park’s Nucleotide Precursor							
UDP-MurNAc-[^14^C]pentapeptide	Intracellular lipid I synthesis	MraY	Enzymatic studies	NA	*E. coli*	Purified MraY produced Lipid I	(97, 99)

Lipid Precursor							
[^14^C]GlcNAc, lipid II	Extracellular transglycosylation/transpeptidation	TGase	Enzymatic studies	NA	*E. coli*	PBP1A is a key enzyme in peptidoglycan synthesis	(104)

Glycan Core Precursors							
[^3^H]acetylglucosamine	Intracellular transglycosylation	MurG	Uptake studies	NA	*E. coli*	Rapid bacterial uptake	(93)
[^18^F]fluoroacetamido-d-glucopyranose	Intracellular transglycosylation	MurG	Uptake/competition studies	Murine myositis model infection vs sterile inflammation ([^18^F]FAG vs [^18^F]FDG)	*E. coli*	Clearly visualized infections, not inflammations	(112)

a Abbreviations: RCY = radiochemical yield; *E. coli* = *Escherichia coli* Gram-negative; *S. aureus* = *Staphylococcus aureus* Gram-positive; NA = not applicable.

### 3.1 Amino Acids-Based Probes

Amino acids are required for several biochemical processes in mammalian and bacterial cells, with the latter showing more preference for d-amino acids than l-amino acids.^60^ A decade of research has been dedicated demonstrating the capability of the bacteria to utilize exogenous d-amino acids for peptidoglycan biosynthesis.^61^ This process takes place either in the periplasm by an exchange of d-amino acid at the terminal d-Ala of the peptide stem or in the cytoplasm via *de novo* synthesis of peptide precursors, facilitated by ld-transpeptidase and d-alanine-d-alanine ligase (Ddl) activity, respectively^59,62−65^ (Figure 2).

**Figure 3:**
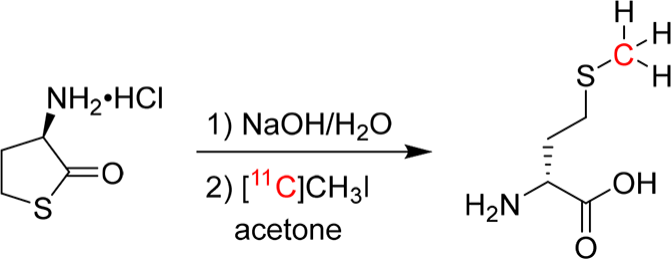
Radiosynthesis of d-methyl-[^11^C]methionine. Reproduced with permission from ref (66), published 2017 under a Creative Commons Attribution 4.0 International License.

Neumann et al.^66^ characterized *in vitro* bacterial uptake of d-[^14^C]methionine (d-Met), d-[^14^C]valine (d-Val), and d-[^14^C]phenylalanine (d-Phe), and significantly more cell incorporation was seen with d-[^14^C]Met for both *E. coli* (Gram-negative) and *S. aureus* (Gram-positive) bacteria. These results concur with those observed in another study by Kwak.^67^ The possible explanation for the 6–10-fold higher accumulation over d-Val/d-Phe might be because the metabolism of d-Met is independent of the bacterial growth rate.^63^ Based on this finding, the group further explored d-Met synthesized by reacting [^11^C]CH_3_I with a d-homocysteine thiolactone precursor, for PET/CT imaging using mice bearing *E. coli* and *S. aureus*(66) (Figure 3). High uptake of d-methyl-[^11^C]methionine (d-[^11^C]Met) was reported in both Gram-positive (0.96%ID/cc) and Gram-negative (0.78%ID/cc) bacteria strains, with significant reduction obtained by co-incubation with unlabeled d-Met, suggesting that incorporation was specific to activate the metabolic pathway. The PET/CT imaging of d-[^11^C]Met showed >2.5-fold higher counts and selectivity for infection over inflammation using murine myositis model in comparison to l-[methyl-^11^C]methionine (l-[^11^C]Met). Non-specific d-[^11^C]Met accumulation was observed in the lungs and the respiratory and gastrointestinal tracts.^66^

**Figure 4:**
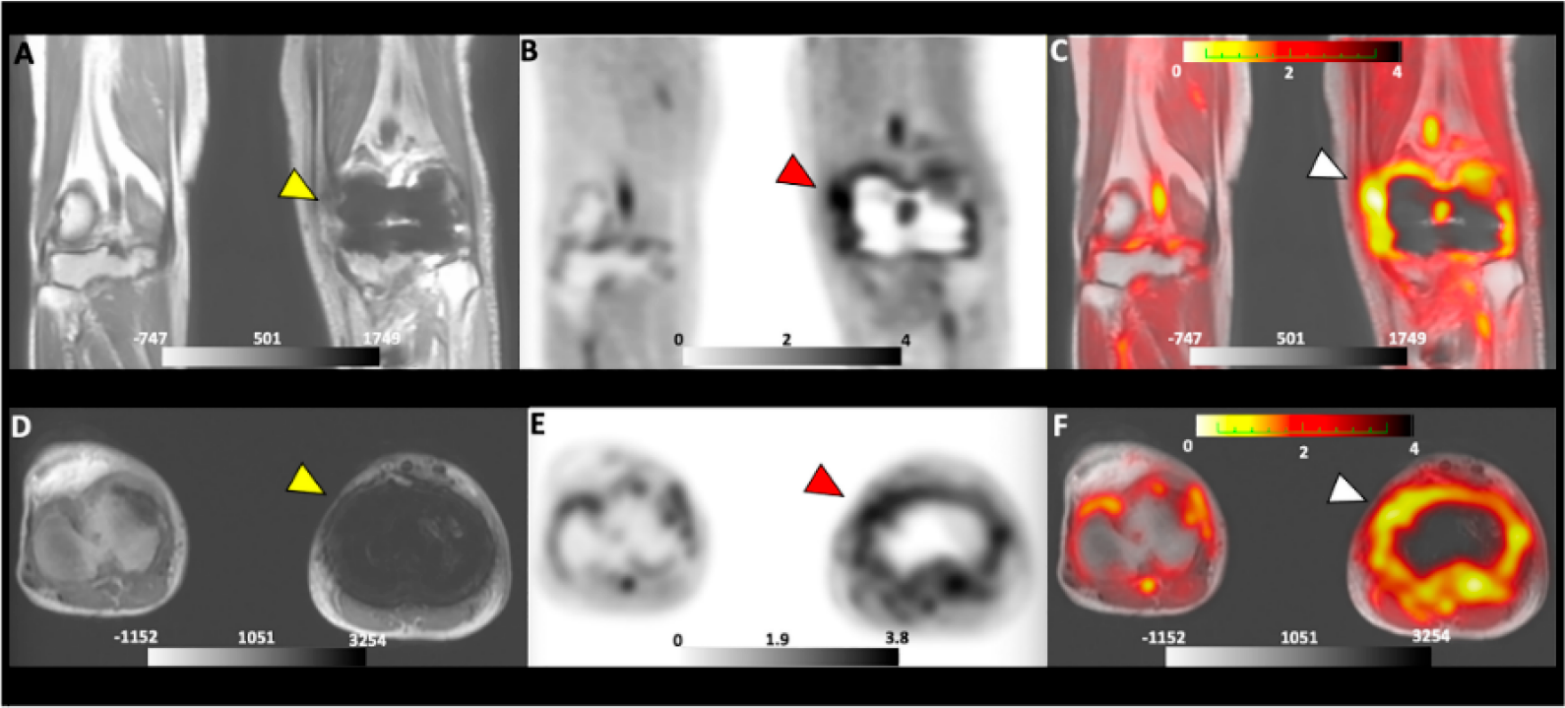
PET/MRI images of d-[^11^C]Met of a patient with suspected PJI. The yellow arrow indicates the MRI scan (A and D). Red and white arrows indicate the localization of d-[^11^C]Met in the infected joint area by using PET (B and E) and PET/MRI (C and F), respectively. Reprinted with permission from ref (70), published 2022 under a Creative Commons Attribution 4.0 International License.

Subsequently, the group of Stewart et al.^68^ optimized the radiosynthesis yield of d-[^11^C]Met using d-homocysteine precursor, and specific uptake was observed in various Gram-negative and -positive pathogens. Similarly, Muranaka et al.^69^ showed superior detection sensitivity of *S*-methyl-[^3^H]-d-methionine (d-[^3^H]Met) with >2-fold higher infection/background ratio over [^18^F]FDG using a lung infection model in mice. Based on these findings, Polvoy et al.^70^ successfully reported the first clinical translation of d-[^11^C]Met in prosthetic joint infections (PJIs), and the data indicated significant (*p* < 0.0001) focal infection localization with low background signal using PET/MRI scan (Figure 4). Although the results are promising, the probe application might be limited to musculoskeletal infections due to the high background signal observed in soft tissues. Following the positive prospects of d-[^11^C]Met in a clinical setting, d-[^18^F-CF_3_]-methionine was developed to mitigate the logistics challenges associated with the shorter half-life of the [^11^C]. Despite the bacterial uptake assays not being performed, the low radiochemical yield obtained necessitates further optimization.^71^

**Figure 5:**
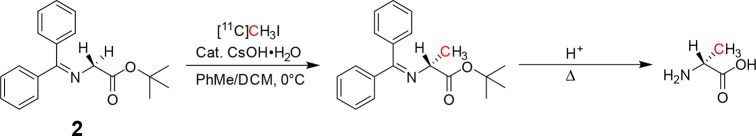
Radiosynthesis of d-3-[^11^C]alanine. Reproduced with permission from ref (72). Copyright 2020 American Chemical Society.

**Figure 6:**
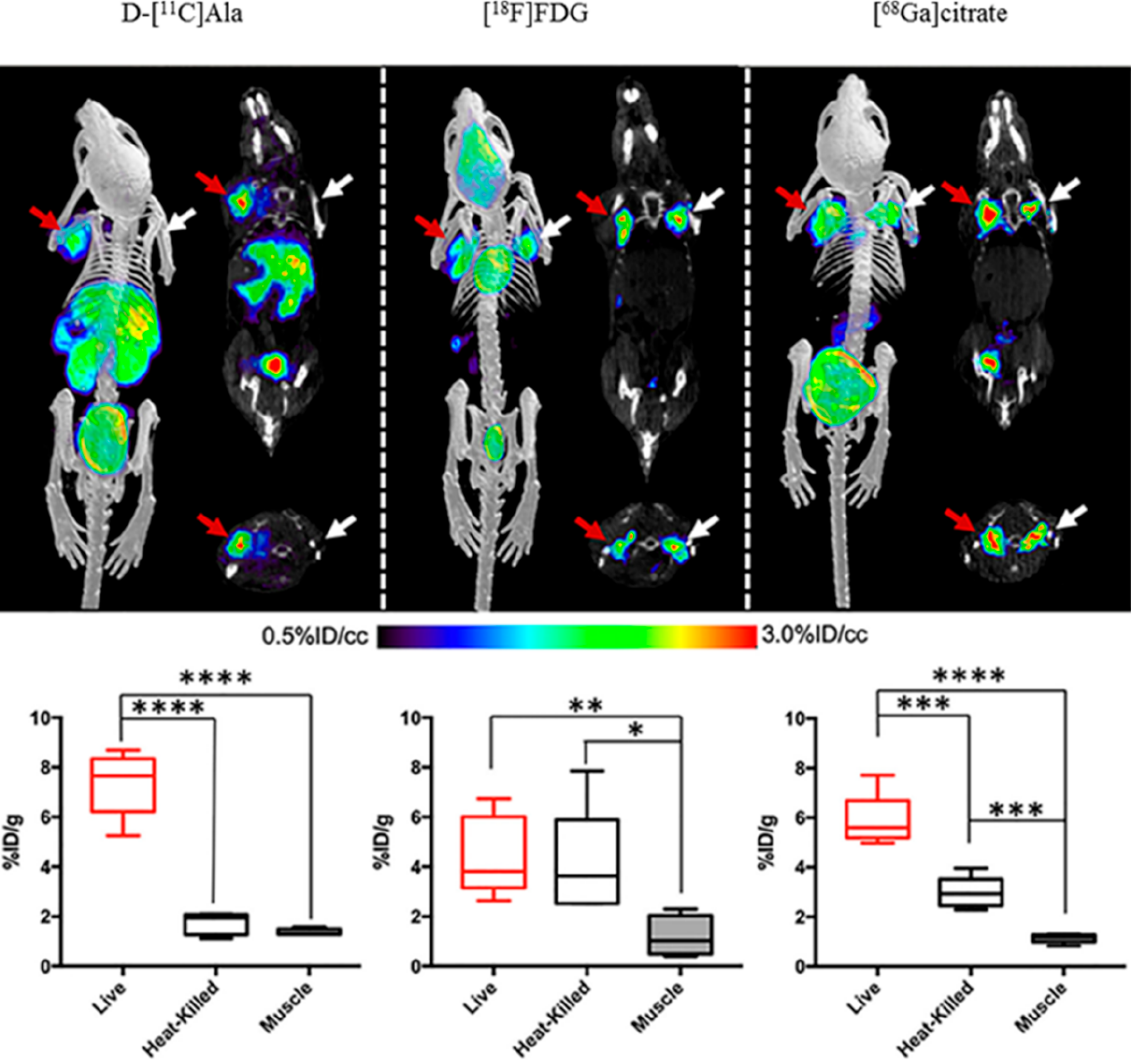
PET/CT biodistribution and corresponding *ex vivo* tissue data of d-[^11^C]Ala, [^18^F]FDG, and [^68^Ga]citrate in an acute infection mouse model inoculated with live bacteria (red arrows) and heat-killed bacteria (white arrows). Reprinted with permission from ref (72). Copyright 2020 American Chemical Society.

The same group synthesized d-3-[^11^C]alanine (d-[^11^C]Ala) through the alkylation of a glycine-derived Schiff-base precursor with [^11^C]methyl iodide (Figure 5). Bacterial metabolism of d-[^11^C]Ala was reported in different Gram-negative and positive bacterial strains with high *in vivo* sensitivity and selectivity (>3.5-fold %ID/g) obtained in comparison to [^18^F]FDG and [^68^Ga]citrate^72^ (Figure 6). The study further reported possible applications in detecting spinal infections, pneumonia, and infections with antibiotic-resistant strains, which is currently challenging in the clinical setting. Similar *in vitro* results were obtained using d-2,3-[^3^H]Ala. However, the group did not further pursue the *in vivo* biodistribution investigations of the probe.^67,69^ Based on these promising results, for the first time, an ^18^F-labeled alanine derivative (d-[^18^F-CF_3_]alanine) was reported for infection imaging. Unfortunately, the probe performed less than expected, with low specific activity and bacterial incorporation.^71^

Bacteria can also incorporate d-amino acids with large aromatic substituents, including d-phenylalanine (d-Phe), d-tyrosine (d-Trp), and d-tryptophan (d-Tyr), as substrates for transpeptidation.^59,62^ Similarly,^66^ Kwak^67^ investigated the bacterial uptake of d-[^14^C]Phe, which showed specific uptake despite its lower rate compared to d-[^14^C]Met using *E. coli*. However, this study further synthesized (*R*)-2-amino-3-(4-[^18^F]fluorophenyl)propanoic acid, an analog of d-Phe by substitution of a proton with [^18^F], to test the changes in bacterial uptake studies.^73^ According to the results, the replacement of ^14^C with ^18^F did not affect the uptake of d-Phe, and a significant specificity (>14-fold higher than [^18^F]FDG) was observed in *E. coli* cultures. The study demonstrated the feasibility of d-[^18^F]Phe in bacteria-specific targeting, which warrants further investigation in mouse infection models and PET/CT imaging.

**Figure 7:**
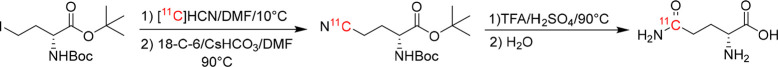
Radiosynthesis of d-5-[^11^C]glutamine. Reproduced with permission from ref (75). Copyright 2021 American Chemical Society.

**Figure 8:**
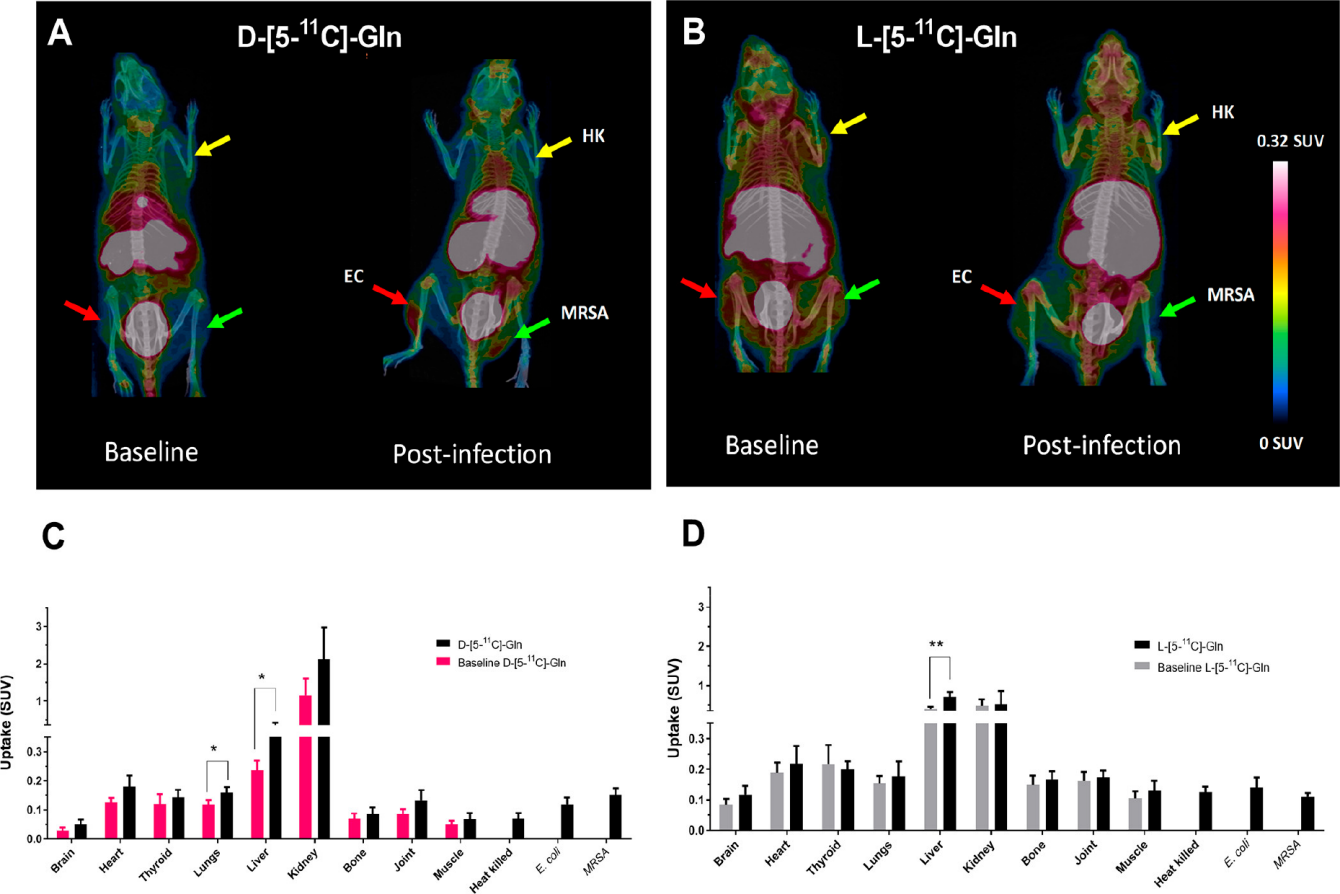
PET/CT scans with corresponding *ex vivo* biodistribution of d-5-[^11^C]Gln (A and C) vs l-5-[^11^C]Gln (B and D) in healthy baseline and infected mice: inoculated with live methicillin-resistant *S. aureus* (MRSA) (green arrow), *E. coli* (EC) (red arrow), and heat-killed bacteria (HK) (yellow arrow). Reprinted with permission from ref (75). Copyright 2021 American Chemical Society.

In many Gram-positive strains, d-glutamate is converted to d-glutamine (d-Gln) at glutaminase’s second position of the peptidoglycan precursor.^74^ Kwak^67^ reported high bacterial uptake of d-[^3^H]glutamate, blocked by co-administration of unlabeled glutamate, indicating non-specific uptake. A similar experimental design was used in another study by Renick et al.,^75^ utilizing d-5-[^11^C]glutamine (d-5-[^11^C]Gln) synthesized by a two-step approach by reacting *tert*-butyl-2-((*tert*-butoxycarbonyl)amino)-4-iodobutanoate with [^11^C]HCN, followed by deprotection of the nitrile intermediate (Figure 7). The *in vitro* investigations of d-5-[^11^C]Gln showed high uptake in live methicillin-resistant *S. aureus* and *E. coli*, which was inhibited with increasing concentrations of unlabeled reference (Figure 8). The PET/CT image-guided tracer biodistribution using a dual myositis mouse model showed 1.64-fold higher infection-to-background ratios for both Gram-positive and -negative bacteria. Unlike l-5-[^11^C]Gln, the d-5-[^11^C]Gln signal also allows for the differentiation of the infection from inflammation (induced heat-killed bacteria).

In addition, investigations of PBP enzymatic reactions were conducted using unlabeled lipid II and d-amino acids. PBP recognized the d-enantiomers and not the l-enantiomers, suggesting that the reaction occurs at the amines in the alpha-amino group and not the epsilon-HH_2_ moiety, with inhibitory effects observed with the addition of β-lactams.^59,62,76,77^ Interestingly, previous studies have reported the potential of bacteria cells to make use of l-amino acids as the precursors for cell wall targeting, with uptake observed with l-2,3-[*S*-methyl-^3^H]methionine (l-[^3^H]Met), l-[^3^H]alanine (l-[^3^H]Ala), l-2,3,4-[^3^H]glutamic acid (l-[^3^H]Glu), l-2,5-[^3^H]histidine (l-[^3^H]His), and l-3-[^11^C]alanine (l-[^11^C]Ala).^68,69,78^ Contrasting results were obtained in another study with [^3^H]-l-Met, with no accumulation observed at the infected site, and this discrepancy is hypothesized to be due to the use of different bacterial strains.^66,69^ This non-stereoselectivity is due to the ability of bacteria to convert l-amino acids to d-amino acids intracellularly using the amino acid racemase pathway for metabolic incorporation into the peptidoglycan. This hypothesis was supported by a previous study that showed a significant accumulation of intracellular l-alanine pools over d-amino acids in cells treated with d-alanine racemase alanine antibiotic (d-cycloserine).^79^ However, experimental studies are required to validate peptidoglycan metabolic incorporation of radiolabeled l-amino acids in bacteria.^80−83^ Despite the results, biodistribution studies using mouse infection models demonstrated similar target-to-non-target (T/NT) ratios of ∼1 for both l- and d-amino acids, therefore limiting the sensitivity of PET/CT diagnostics for bacterial infection.^75,78^

d-Amino acids are incorporated through the cytoplasmic pathway mediated by Ddl and MurF enzymes.^84^ Alternatively, Kuru et al.^85^ proposed periplasmic transpeptidase as the main pathway of d-amino acids incorporation in the peptidoglycan, with reduced fluorescence intensity observed in β-lactams and d-cycloserine (DCS)-treated bacteria including l- and d-TP mutant strains. Moreover, this study further disputes the cytoplasmic pathway metabolism with a persisting fluorescence intensity observed for wild-type and Ddl mutant cells. Based on these findings, the possibility of one- or two-way pathway involvement in bacterial cell incorporation of d-amino acids is substantiated.

Overall, several pre-clinical studies have demonstrated the feasibility of radiolabeled d-amino acids as PET/CT infection imaging agents with prospects for clinical translation. However, one concern noted is the observed active metabolism by the microbiome and other non-infected organs, which might result in data misinterpretation. A limitation would also be the ability of fungi to utilize d-amino acids in several metabolic activities, including carbon and nitrogen absorption.^86,87^ In particular, d-amino acid-based PET tracers such as d-5-[^11^C]Gln have been shown to image fungal infection with high specificity.^88^ Consequently, the ability of the relevant tracers to differentiate bacterial infections from fungal infections in the clinical setting would be compromised. In addition, the development of ^18^F-labeled d-amino acids is hampered by poor radiochemical yield and possible defluorination *in vivo*.^71,89^ Despite the mentioned limitations, the reported tolerance of ld-transpeptidase to different modifications at the C-terminal creates an opportunity to design a library of d-amino acid analogs with more optimized radiosynthesis and a favorable output of primary pharmacology. Moreover, the differentiation of pathogen- (bacteria vs fungi), bacterial species-, or phenotype-specific PET imaging might be realized, thus improving guided therapy and identifying antibiotic-resistant strains.^72,90,91^

### 3.2 d-Amino Acid Dipeptide-Based Probes

**Figure 9:**
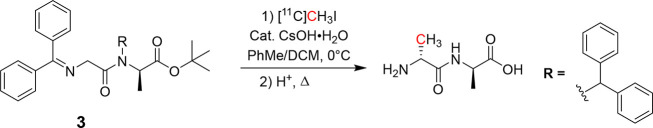
Radiosynthesis of d-3-[^11^C]Ala-d-Ala. Reproduced with permission from ref (72). Copyright 2020 American Chemical Society.

During peptidoglycan biosynthesis, metabolic incorporation of the d-Ala-d-Ala dipeptide at the tripeptide terminal by MurF results in a pentapeptide chain (lipid I), which is ultimately incorporated into the existing peptidoglycan chain. This early-stage pathway has been targeted for cell wall imaging using a fluorescent d-Ala-d-Ala dipeptide analog.^85^ The successful incorporation of d-Ala-d-Ala also requires binding compatibility with the MurF enzyme, which has high specificity for the C-terminal residue. This was demonstrated by the previous work with high bacterial uptake of fluorescence-tagged d-amino acid dipeptide modified at the N-terminus compared to C-terminal-modified probes reported.^85,92^ By adapting the same labeling strategy, Parker et al.^72^ developed a d-amino dipeptide for cell wall targeting by successfully synthesizing a d-3-[^11^C]alanyl-d-alanine (d-[^11^C]Ala-d-Ala) probe via ^11^C-alkylation of the N-terminus of a d-Ala-d-Ala glycine Schiff precursor followed by deprotection of the amine groups (Figure 9). The *in vitro* screening of d-[^11^C]Ala-d-Ala showed uptake of 9 Bq/10^6^ cfu in *E. coli*, which was nearly quantitatively inhibited in heat-killed culture sample. In addition, a partial *in vitro* screen using d-[^11^C]Ala-d-Ala showed uptake levels ranging from 5 to 35 Bq/10^6^ cfu in several other bacteria, which justifies further evaluation of *in vivo* imaging by PET/CT.

In another study by Goodell,^93^ a [^3^H]A2pm-d-Ala dipeptide was incorporated into the bacterial cell wall at a slow rate. This might explain the lower uptake of d-[^11^C]Ala-d-Ala in comparison to d-[^11^C]Ala reported in a previous study.^72^ The study suggested periplasmic degradation of the dipeptide into A2pm and d-Ala as substrates of carboxypeptidase before transportation into the cytoplasm for re-integration into the downstream pathway by the Dld enzyme. This indicates a limit of d-amino acid dipeptide-based probes in cell wall targeting due to possible reduction in labeling signals, also reported previously for a fluorescent-based tracer with the addition of peptidoglycan-digesting enzyme (lysozyme).^92^ Unfortunately, bacteria have a perpetual built-in resistance to β-lactam-based antibiotics by substituting terminal d-alanine with a variety of d-amino acids, including d-lactate and d-serine.^94,95^ Therefore, to address the mentioned limitation, Filp et al.^96^ have reported the synthesis of a variety of ^11^C-labeled d-amino acid dipeptides substituted with different amino acids at the terminal residue, which could indeed preserve the probes from degradation by carboxypeptidases. However, efforts should be made to evaluate these dipeptide derivatives’ efficacy in bacterial targeting and determine the value of PET imaging.

### 3.3 Park’s Nucleotide and Lipid-Based Probes

The formation of lipid I is the initial membrane-based step of peptidoglycan biosynthesis facilitated by MraY transferase.^97,98^ Previous studies used chemo-enzymatically synthesized UDP-MurNAc-l-[^14^C]Ala-γ-d-Glu-*meso*-A2pm/Lys-d-Ala-d-Ala (UDP-MurNAc-[^14^C]pentapeptide) to study the biochemical properties of this enzyme. They demonstrated the MraY selectivity for UDP-MurNAc-pentapeptide analogs during peptidoglycan synthesis, with high specificity observed toward nucleotide substrates with alanine as opposed to glycine in positions 1 and 4 rather than in position 5.^99−101^ In particular, UDP-MurNAc-pentapeptides labeled with ^14^C at the l-Ala/A2pm position in the presence of MraY led to the formation of lipid I.^100^ Subsequently, other studies showed that UDP-MurNAc-pentapeptide derivatives modified with fluorescein at the *meso*-diaminopimelic acid (m-DAP) residue were more efficiently incorporated into the cell wall of Gram-positive bacteria compared to Gram-negative bacteria.^102,103^ This evidence strongly supports the further investigation of ^14^C-labeled UDP-MurNAc-peptide probes for specific targeting, with possible differentiation of infections caused by Gram-positive and -negative strains, although PET/CT-suitable probes using ^18^F and ^11^C have not been reported yet.

A further addition of lipid II to the existing murine chain generally occurs in the periplasmic space, facilitated by PBP. Born et al.^104^ and Bertsche et al.^105^ used the *in vitro* murine synthesis assay to study the transglycosylation and transpeptidation reactions which catalyze the cross-linking of glycan and peptide chains, respectively, using an enzymatically produced [^14^C]GlcNAc-labeled lipid II as PBP substrate. Consequently, the catalytic recognition and turnover of [^14^C]GlcNAc-labeled lipid II by PBP may be an interesting strategy for bacterial characterization *in vivo*, which has not yet been reported. Of note, Sadamoto et al.^102^ attempted this approach in live bacteria using fluorescently labeled lipid I/II derivatives; unfortunately, the cells did not take up these probes. The group hypothesized that the lack of uptake might be due to the low affinity of glycosyltransferases (TGase) toward lipid II analogs, with shorter lipid chains affecting transglycosylation,^106^ or due to the high molecular weight (>1000 g/mol) of these derivatives preventing extracellular membrane permeability. As the intra- and extracellular entrapment of precursor lipids I/II is a key step in peptidoglycan synthesis,^43^ the latter findings might deter translation into nuclear infection imaging. Park’s nucleotides and lipid-derived substrates in bacteria-targeting research are mainly limited due to the complex synthetic processes involved in developing structurally similar compounds. However, newly developed chemical and enzymatic synthesis methods have opened up the opportunity to design precursors with different modifications for the possible incorporation of radioisotopes. Therefore, further exploration in bacterial uptake studies using Gram-positive strains is required to assess their role and value for infection imaging.^107,108^

### 3.4 Carbohydrate/Glycan Core-Based Probes

**Figure 10:**
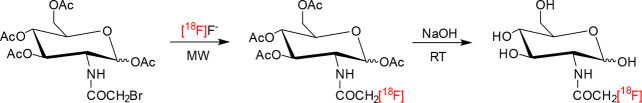
Radiosynthesis of 2-deoxy-2-[^18^F]fluoroacetamido-d-glucopyranose. Reproduced with permission from ref (112). Copyright 2011 Elsevier Inc.

**Figure 11:**
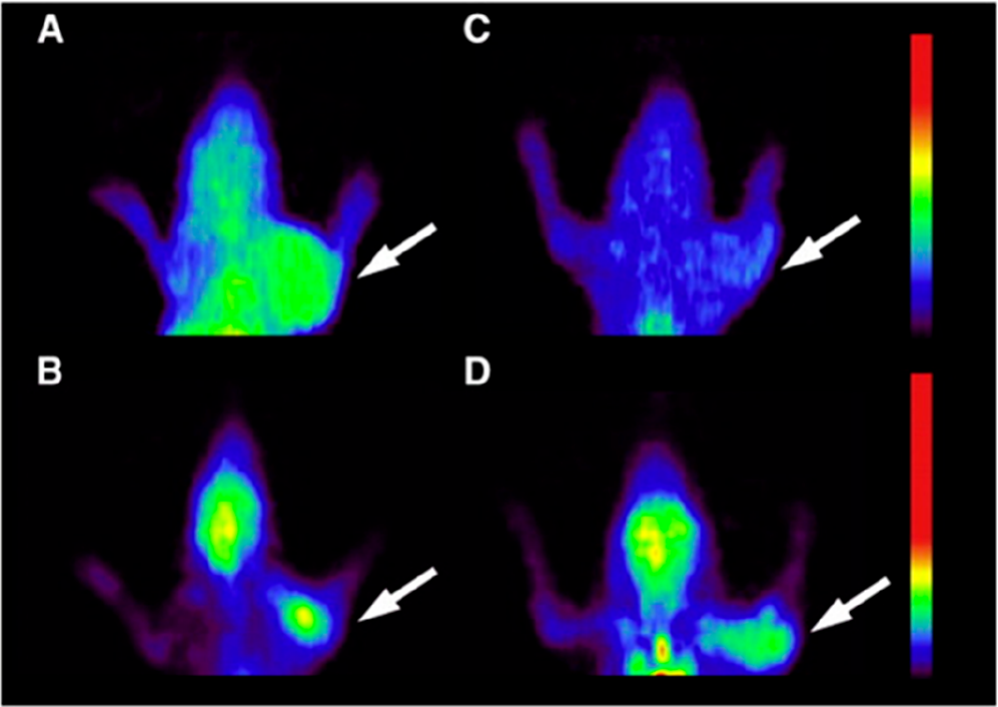
PET localization of [^18^F]FAG and [^18^F]FDG in rats bearing bacterial infection or sterile inflammation. The white arrows indicate the accumulation of [^18^F]FAG (A and C) and [^18^F]FDG (B and D) at the sites of infection (A and B) and inflammation (C and D). Reproduced with permission from ref (112). Copyright 2011 Elsevier Inc.

Both *N*-acetylmuramic acid (MurNAc) and *N*-acetylglucosamine (GlcNAc) are the first amino sugar substrates of the peptidoglycan biosynthesis, forming the backbone of the polymer chain. Most bacteria are capable of recycling exogenous GlcNAc and MurNAc substrates through phosphorylation by cytoplasmic kinase MurK, resulting in GlcNAc-6-phosphate for initiation of peptidoglycan biosynthesis, cell wall macromolecules (lipopolysaccharides, teichoic acids), or catabolic pathway of glycolysis.^109−111^ Rapid uptake and integration of [^3^H]GlcNAc into peptidoglycan were reported in a previous study.^93^ A follow-up study by Martínez et al.^112^ synthesized an *N*-acetyl-d-glucosamine analog for bacterial uptake and imaging for the first time (Figure 10). This study used ^18^F-labeled 1,3,4,6-tetra-*O*-acetyl-2-deoxy-2-bromoacetamido-d-glucopyranose followed by hydrolysis to synthesize 2-deoxy-2-[^18^F]fluoroacetamido-d-glucopyranose ([^18^F]FAG). The *in vitro* and *in vivo* uptake experiments using *E. coli* showed significant incorporation of [^18^F]FAG compared to its counterpart [^18^F]FDG, which made it possible to distinguish bacterial infection from sterile inflammation (with a T/NT of 1.68) by PET/CT imaging (Figure 11).

A study by Hu et al.^113^ revealed that hydroxyl groups are essential for binding GlcNAc to MurG. Therefore, the ^18^F-acetyl conjugation strategy did not affect the binding properties of [^18^F]FAG. This might be due to the acetyl group’s inability to participate in the binding interface of MurG, as previously reported, allowing the synthesis of [^18^F]FAG-containing lipid II for ultimate incorporation into peptidoglycan; however, more work is required to solidify the route of incorporation.^114,115^ Despite the promising results, the probe showed non-specific hepatic and pulmonary uptake. Carroll et al.^116^ developed a library of ^18^F-labeled glucosamine derivatives using different prosthetic groups. However, the probes showed unfavorable pharmacokinetics, including elevated background signals and *in vivo* defluorination. To address these shortcomings, Sadamoto et al.^108^ synthesized and demonstrated bacterial uptake of GlcNAc-1-phosphate derivatives modified with a ketone at the *N*-acetyl position, which presents an attractive alternative to reduce the background noise. Although the hydroxyl groups are required for recognition of [^18^F]FAG by MurG, the study reported significant association with lactobaccilli of Alexa-Fluor-488-acetylated glucosamine derivatives due to increased hydrophobicity, followed by the intracellular removal of the *N*-acetyl group for ultimate incorporation into the cell wall.^108^ As a result, this supports the investigation of 1,3,4,6-tetra-*O*-acetyl-2-deoxy-2-[^18^F]fluoroacetamido-d-glucopyranose as a potential glucosamine derivative for cell wall targeting, which was not previously evaluated.^112^

A novel recycling pathway unique to *Pseudomonas putida* involving direct integration of UDP-MurNAc into *de novo* synthesis without requiring UDP-GlcNAc as a substrate has been documented.^117^ This was demonstrated by direct bacterial cell wall incorporation of MurNAc derivatives fluorescently labeled at the *N*-acetyl termini using click chemistry;^118^ however, no research has been attempted to develop radioactive MurNAc derivatives for direct *in vivo* diagnostic imaging. Unlike [^18^F]FAG which is metabolized by mammalian cells,^119^ a MurNAc-based radiolabeled probe will potentially increase the specificity and selectivity of the bacterial foci, as it is unique to bacteria. A plausible radiolabeling strategy could be explored with tolerated modifications at the *N*-acetyl terminus of the MurNAc residue using ^18^F- or ^11^C-labeling, enabling targeting and imaging of peptidoglycan biosynthesis using PET/CT.^118^

### 3.5 Oligopeptide-Based Probes

One essential pathway of peptidoglycan biosynthesis is the integration of oligopeptides shed from the existing polymer chain into the cytosol to be re-used in the formation of the *de novo* peptidoglycan polymer chain. To understand this pathway, Goodell^93^ reported uptake in *E. coli* for the l-Ala-d-Glu-*meso*-A2pm-tripeptide and the l-Ala-d-Glu-A2pm-d-Ala-tetrapeptide labeled at the third position with 3,4,5-[^3^H]A2pm and demonstrated the enzymatic conversion into UDP-MurNAc-tri- and pentapeptides for ultimate re-integration into the peptidoglycan chain. The study reported degradation of the tetrapeptide at the terminal residue, resulting in the tripeptide and d-Ala, suggesting that the tripeptide was responsible for labeling the cell wall. In a follow-up study, Olrichs et al.^120^ developed an l-alanine-γ-d-glutamine-l-lysine-tripeptide fluorescently labeled with *N*-7-nitro-2,1,3-benzoxadiazol-4-yl (AeK-NBD) at the lysine terminal and demonstrated that peptidoglycan incorporation was not affected by the replacement of the A2pm at position 3 with Lys. The study further showed that recycling depends entirely upon substrate recognition by Mpl, with a high specificity reported for the tri- or tetrapeptide composed with A2pm/l-Lys at position 3. Furthermore, substrate incorporation was inhibited in Mpl mutant bacteria.^121,122^ Taking the observations mentioned above into account, l-Ala-d-Glu-*meso*-A2pm/Lys and l-Ala-d-Glu-A2pm-d-Ala are promising substrates for bacteria-specific targeting and imaging; however, their radiopharmaceutical development and value for PET imaging of infection are outstanding.

## 4 Design Approach and Challenges for Translation

Developing and validating new radiotracers for more specific imaging of infection are commons goal to improve diagnosis and health care in patients. Recently, a comprehensive review was published including consensus results, expert opinions, and recommendations regarding the current research standards aiming to improve radiotracers for infection imaging.^33,124^

### 4.1 Precursor Design

Several factors must be considered when developing radioactive probes for specific targeting, including the feasibility of synthesizing a precursor and/or its analogs, the radiosynthesis strategy, and the *in vitro* and *in vivo* validation strategy.^125^ In particular, tracking the peptidoglycan biosynthesis for bacterial targeting requires periplasmic or intracellular metabolism of precursors of interest by a series of enzymes for incorporation into the cell wall. Structural changes such as choice of modification site, molecular size, conjugation of radioisotopes, linker length, or geometry may affect the metabolic pathway and level of compound incorporation.^126,127^ Interestingly, most peptidoglycan-based radioactive precursors reported here performed excellently, differentiating between infection and inflammation. However, a high background signal reported with most radiotracers remains a concern and can compromise translation to routine clinical applications due to the high probability of false-positive outcomes. Therefore, optimization of the biodistribution profile using different structural modifications needs to be explored. It is worth mentioning that the lengthy and complex chemical and chemo-enzymatic procedures during the synthesis of peptidoglycan-based precursors leave a small room for structural manipulations.^37,128,129^ A plausible solution to mitigate these challenges and fast-track the development process requires the use of computational tools such as docking and high-throughput screening to create chemical libraries with diverse molecular adaptations that will predict how structural, chemical, physical, and physicochemical properties of molecules can alter the net cellular uptake through interaction with target enzymes and the pharmacokinetic profiling.^127,130−133^

### 4.2 Radiolabeling Approach

Another critical factor in the design process hinges on the choice of radioisotope (Table 2), which is influenced by the metabolic pathway of interest (extracellular vs intracellular).^134,135^ Often radiolabeling methods include radio-metals such as ^68^Ga, ^64^Cu, or ^111^In, which may require chemical modifications by introducing bifunctional chelators such as 1,4,7,10-tetraazacyclododecane-1,4,7,10-tetraacetic acid (DOTA) and 1,4,7-triazacyclononane-1,4,7-triacetic acid (NOTA) for complexation of the precursor and radioisotope.^125,132,136^ As a result, an ideal radiolabeling strategy in peptidoglycan biosynthesis targeting should preserve both the chemical structure and targeting moiety of the precursor.^137^ In this case, direct labeling using radioisotopes with long half-lives, i.e., ^3^H or ^14^C, is abundantly used for uncovering peptidoglycan biosynthesis and screening for potential precursors using enzymatic and bacterial uptake methods.^32^ For application in PET/CT imaging, replacement with non-metallic radioisotopes, including ^11^C and ^18^F, has minimal influence on metabolic uptake and incorporating the peptidoglycan-based precursors.^67,73,96^ These short-lived radioisotopes can present challenges during radiosynthesis, including the use of harsh conditions, which often result in unwanted byproducts and compromise the stability of the precursor. Therefore, the use of click chemistry with different prosthetic groups may be an alternate radiosynthesis approach, especially applicable to ^18^F or ^123^I; however, this remains practically unexplored in comparison to ^11^C.^73,138−140^

**Table 2:** Properties of Commonly Used SPECT and PET Radioisotopes^125,135,141^

Isotope	Physical half-life, *t*_1/2_ (h)	Decay mode^a^	Decay energy (MeV)	Production	Radiolabeling strategy
^68^Ga	1.1	β–, EC, γ	1.899	Generator	Chelation
^64^Cu	12.7	EC, β+	0.653	Cyclotron	Chelation
^18^F	1.8	β+, EC	0.634	Cyclotron	Direct
^123^I	13.2	γ, EC	0.159	Cyclotron	Direct
^11^C	0.3	β+	0.960	Cyclotron	Direct
^111^In	67.2	γ, EC	0.245	Cyclotron	Chelation
^99m^Tc	6.0	γ	0.141	Generator	Chelation/Direct

a EC = electron capture.

Click chemistry is a promising approach for *in vivo* pre-targeting of infections and has been well-demonstrated with optical imaging.^36^ This procedure involves a two-step labeling strategy which requires pre-targeting of the peptidoglycan using endocyclic nitrone-, alkyne-, or azide-modified d-amino acids derivatives, followed by subsequent visualization with a complementary fluorescent reporter using bioorthogonal reactions.^142,143^

**Figure 12:**
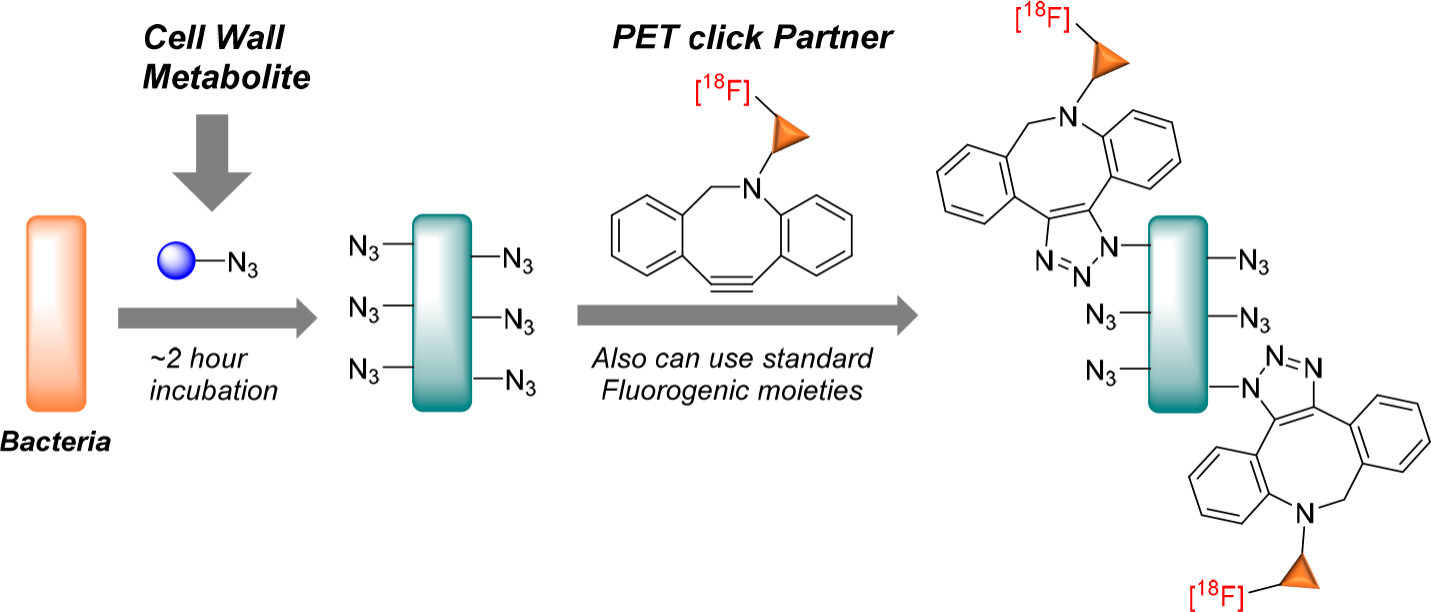
Click chemistry for pre-targeting radiolabeling approach for bacteria using d-azidoalanine (cell wall metabolite) followed by [^18^F]cyclooctyne (PET “click” partner). Reproduced with permission from ref (123). Copyright Alanizi, 2021.

Interestingly, the Wilson group pioneered using ^18^F-labeled click chemistry with the peptidoglycan-based targeting azide-modified d-alanine derivative for the first time (Figure 12). This promising metabolic targeting approach has set a footprint for applying click chemistry in PET imaging of infection^123^ and beyond.

### 4.3 *In Vitro* Testing

Further testing of candidate radiotracers should be carried out to investigate the effect of precursor modifications and radioisotopes on pharmacokinetics and cell wall incorporation. *In vitro*, bacterial uptake studies using live vs heat-inactivated cultures were reported for several of the reviewed peptidoglycan precursors. However, uptake does not necessarily prove binding selectivity and accurate representation of the peptidoglycan biosynthesis status. Therefore, competition studies with an unlabeled compound are required as the standard test in most *in vitro* studies.^36^ The latter might not be sensitive enough to elucidate the proposed mechanism of integration since bacteria can use most precursors, including the d-amino acids and glycans, as building blocks in other structures (proteins, lipopolysaccharides, teichoic acids, and nucleic acids) or as a source of energy.^144,145^ As stated in the previous section, using radioisotopes with a long half-life may be beneficial for the *in vitro* evaluation of various precursors with different structural modifications. This setting will require the integration of advanced techniques with high sensitivity, such as polyacrylamide high-pressure liquid chromatography (HPLC), polyacrylamide gel electrophoresis, autoradiography, mass spectrometry, and nuclear magnetic resonance (NMR).^37,104,146−148^ Once potential derivatives specific to the peptidoglycan metabolism have been identified, they can be optimized for ^18^F- and ^11^C-radiosynthesis, followed by *in vitro* uptake using bacterial strain mutants of the enzymes involved in the metabolic process of interest as negative controls. This can be achieved through genetic manipulation or the use of targeted peptidoglycan inhibitors, such as antibiotics.^36,40,85^ Also, for specific *in vitro* tests, the radioactive compounds should be evaluated using planktonic bacterial cultures, as in the clinical setting persistent or antimicrobial-resistant bacteria are often hidden in biofilms. This will improve the likeliness of translating results from pre-clinical evaluations further into the clinics.^149^

### 4.4 *In Vivo* Evaluation

Imaging infectious processes and discriminating them from sterile inflammation are still issues in diagnostic imaging.^33^ However, small radiolabeled molecules with high selectivity and specificity for bacteria emerged with great potential as precise agents for non-invasive infection imaging. Despite that, their clinical translation has been limited by access and suitability to appropriate animal infection models as well as the study design that is allowed based on animal pathology or well-being.^150−152^ The variability in the choice of animal model and study design has been observed across different studies, which might pose a challenge to data interpretation and reproducibility. For example, a previous study reported bacterial uptake of [^3^H]-l-Met using lung infection models which was not observed in a study using a murine myositis model.^66,69^ This inconsistency could be due to a number of factors, including the use of different bacterial strains, the number of colonies inoculated, the type of animal infection model, or the imaging time points chosen during the course of infection. Therefore, to successfully provide valid pre-clinical data for clinical translation, the animal model should be standardized and reflect the pathogenesis of infectious disease in humans. Thus, further harmonizing the design and animal study requirements is recommended.^151,153^ Furthermore, it should be considered that targeting bacteria with low metabolic activity will probably not render sufficient tracer “load” to allow for its visualization. Therefore, the correct window to employ imaging to detect or monitor infection should be considered.^78^ Of note, antibiotics also interfere with bacterial cell wall synthesis drastically and hamper the uptake of these tracers. Inherently challenging will be detecting infections by intracellular bacteria such as *Mycobacterium tuberculosis*, *Salmonella typhimurium*, *Listeria monocytogenes*, and *Legionella pneumophila*.^154^

## 5 Conclusion and Future Directions

Enzymatic studies and functional fluorescence-based imaging have shed bright light on peptidoglycan biosynthesis and led to innovative translation of peptidoglycan-based drugs or precursors for molecular imaging. However, despite the constant novelty of fluorescence imaging, research into developing and translating radiolabeled peptidoglycan precursors remains a major undertaking, with several limitations. The latter hinges on complex chemical procedures involved in synthesizing molecules of interest, which can restrict the radiotracer design and its adaptation with various modifications. Given the strict peptidoglycan assembly processes which is the target for possible incorporation, the described molecules are limited to radiochemistry involving covalent ^18^F- or ^11^C-substitution, i.e., radioactive tagging with isotopes that require complex chemistry and a cyclotron. As a result, due to progressive limitations and the complexity of the involved infrastructure, most research laboratories may have slowed down their efforts toward furthering development and subsequent clinical translation of new radiotracers for nuclear imaging of bacterial infections. Therefore, strategies to address these challenges should include technological innovation in biology, chemistry, and radiochemistry, which can be fostered through solid collaboration and the advocacy of multidisciplinary research.

## Abbreviations

A2pm: 2,6-diaminopimelic acid
Ala: alanine
AmpD: *N*-acetylmuramyl-l-alanine amidase
C55-P: undecaprenyl phosphate
CT: computed tomography
DAP: 2,6-diaminopimelic acid
Ddl: d-alanine-d-alanine ligase
DOTA: 1,4,7,10-tetraazacyclododecane-1,4,7,10-tetraacetic acid
*E. coli*: *Escherichia coli*
EC: electron capture
FAG: fluoroacetamido-d-glucopyranose
FDG: fluorodeoxyglucose
GlcNAc: *N*-acetylglucosamine
Glu: glutamate
Gln: glutamine
GTase: glycosyltransferase
Gram (+): Gram-positive
Gram (−): Gram-negative
His: histidine
HMPAO: hexamethylpropyleneamine oxime
HPLC: high-pressure liquid chromatography
LAFOV: large axial field of view
LdcA: ld-carboxypeptidase
Lys: lysine
Met: methionine
MIP: maximum intensity projection
MpaA: murein peptide amidase
Mpl: murein peptide ligase
MRI: magnetic resonance imaging
MurA: UDP-*N*-acetylglucosamine enolpyruvyl transferase
MurB: UDP-*N*-acetylenolpyruvoyl glucosamine reductase
MurC: UDP-*N*-acetylmuramoyl-l-alanine ligase
MurD: UDP-*N*-acetylmuramoyl-l-alanine-d-glutamate ligase
MurE: UDP-*N*-acetylmuramoyl-Lalanyl-d-glutamate-2,6-diaminopimelate ligase
MurF: UDP-*N*-acetylmuramoyl-tripeptide-d-alanyl-d-alanine ligase
MurG: *N*-acetylglucosaminyl transferase
MraY: phospho-*N*-acetylmuramoyl-pentapeptide-transferase
MurNAc: *N*-acetylmuramic acid
NMR: nuclear magnetic resonance
NOTA: 1,4,7-triazacyclononane-1,4,7-triacetic acid
PBP: penicillin-binding protein
PCR: polymerase chain reaction
PET: positron emission tomography
Phe: phenylalanine
PJI: prosthetic joint infection
PAGE: polyacrylamide gel electrophoresis
PP: pyrophosphoryl
RCY: radiochemical yield
*S. aureus*: *Staphylococcus aureus*
SPECT: single-photon emission computed tomography
TP: transpeptidase
Trp: tyrosine
Tyr: tryptophan
UBI: ubiquicidin
UDP: uridine diphosphate
US: ultrasound
Val: valine
